# A new method for enhancer prediction based on deep belief network

**DOI:** 10.1186/s12859-017-1828-0

**Published:** 2017-10-16

**Authors:** Hongda Bu, Yanglan Gan, Yang Wang, Shuigeng Zhou, Jihong Guan

**Affiliations:** 1 Department of Computer Science and Technology, Tongji University, 4800 Cao’an Road, Shanghai, 201804 China; 2 School of Computer, Donghua University, 2999 Renming North Road, Shanghai, 201620 China; 3 Shanghai Key Lab of Intelligent Information Processing, and School of Computer Science, Fudan University, 220 Handan Road, Shanghai, 200433 China; 4 The Bioinformatics Lab at Changzhou NO. 7 People’s Hospital, Changzhou, Jiangsu, 213011 China; 5 School of Software, Jiangxi Normal University, 99 Ziyang Avenue, Jiangxi, 330022 China

**Keywords:** Enhancer prediction, Chip-seq, Deep belief network

## Abstract

**Background:**

Studies have shown that enhancers are significant regulatory elements to play crucial roles in gene expression regulation. Since enhancers are unrelated to the orientation and distance to their target genes, it is a challenging mission for scholars and researchers to accurately predicting distal enhancers. In the past years, with the high-throughout ChiP-seq technologies development, several computational techniques emerge to predict enhancers using epigenetic or genomic features. Nevertheless, the inconsistency of computational models across different cell-lines and the unsatisfactory prediction performance call for further research in this area.

**Results:**

Here, we propose a new Deep Belief Network (DBN) based computational method for enhancer prediction, which is called EnhancerDBN. This method combines diverse features, composed of DNA sequence compositional features, DNA methylation and histone modifications. Our computational results indicate that 1) EnhancerDBN outperforms 13 existing methods in prediction, and 2) GC content and DNA methylation can serve as relevant features for enhancer prediction.

**Conclusion:**

Deep learning is effective in boosting the performance of enhancer prediction.

## Background

Eukaryotic gene expression is dominated by a set of events, including chemical modifications to nucleosomes and DNA, the binding of regulatory proteins to DNA and post-transcriptional modifications [1]. Cis-regulatory elements, including enhancers, promoters, insulators and silencers, play the significant role in the process of gene expression. Among them, enhancers are short non-coding DNA sequences that regulate gene expression patterns independent of their relative distance and location to their associated promoter.

Predicting enhancers is important for exploring the biological activities of organisms. Enhancer prediction has moved forward by recent technological advances, including chromatin immunoprecipitation sequencing (ChIP-seq) [2], DNaseI-digested chromatin sequencing (DNase-seq) [3], RNA sequencing (RNA-seq), or Formaldehyde-Assisted Isolation of Regulatory Elements sequencing (FAIRE-seq) [4]. These technical methods enable genome-wide measurement of the structural conformation of DNA, histone modifications and binding sites of regulatory proteins. Furthermore, the FANTOM project [5], ENCODE project [6], and other studies alike focusing on different cell types [7, 8] have massively increased the number of functional genomic data in public [1].

Up to date, several computational methods have been put forward to predict enhancers. For example, support vector machine (SVM) and linear regression models have successfully distinguished novel enhancers active in heart, hindbrain and muscle development [9–11]. Random forests (RFs) [12] have also been trained using histone modifications to predict p300 binding sites in human lung fibroblasts and embryonic stem cells [1]. Two research groups have employed unsupervised approaches based on dynamic Bayesian networks (Segway) [13] and hidden Markov models (ChromHMM) [14]with signatures in ENCODE data to segment the human genome into regions and then assigned potential functions to these regions. However, the unsatisfactory prediction performance and the inconsistency of computational models across different cell-lines call for further exploration in this area.

Here, we proposed a method based on the deep belief network (DBN) for predicting enhancers [15]. We named this new method EnhancerDBN. EnhancerDBN was trained on data from VISTA Enhancer Browser, which contains biologically validated enhancers samples, using three kinds of features consisting of histone modifications, DNA sequence compositional features and DNA methylation. EnhancerDBN turns the prediction problem into a binary classification mission that determines whether any DNA region is an enhancer candidate or not, using a two-step scheme. The first step is to construct a DBN using Restricted Boltzmann Machines (RBMs). The second step is to train and optimise the DBN based deep neural network classifier using the back propagation (BP) algorithm [16]. 10-fold cross validation was employed to evaluate EnhancerDBN. Experimental results indicate that 1) EnhancerDBN can effectively predict enhancers, and outperforms thirteen existing methods, and 2) GC content and DNA methylation are informative for enhancer prediction. Though in bioinformatics area deep learning has also successfully applied to several problems such as drug target prediction [17], to the best of our knowledge, this is the first work that employs deep belief network for enhancer prediction [15].

## Methods

### Datasets

Enhancer data were downloaded from VISTA enhancer Browser (http://enhan-cer.lbl.gov/) on June 1st, 2015, which consist of 741 human enhancers. DNA sequence data and DNA methylation data were the February 2009 assembly of the human genome (GRCh37/hg19). The raw histone modification data were downloaded from NIH Roadmap Epigenomics. A summary of the data used in this paper is given in Table 1.

**Table 1:** Datasets used in this paper

Dataset	g Source	Website
Enhancers	VISTA enhancer Browser	https://enhancer.lbl.gov/
DNA sequence	UCSC	http://hgdownload.soe.ucsc. edu/downloads.html#human
Histone modification	NIH Roadmap	http://www. roadmapepigenomics.org/
DNA methylation	UCSC	http://genome.ucsc.edu/ cgi-bin/hgTables

We used the VISTA Enhancer Browser data because these enhancers were experimentally validated. We chose the histone modification features because some existing works [1, 12] have shown that they are indicative of enhancers. We used GC content for the reason that Erwin et al. [1] found that the heart enhancers were more likely to be identified because they had high GC content. Previous bioresearch also found that low DNA methylation is possibly related to enhancers, which inspired us to use DNA methylation as a type of enhancer features.

We used all the 741 VISTA human enhancers as positive enhancers, and generated 741 negatives by randomly selecting 741 genomic background regions of similar length and chromosome distribution to the positives. As in the existing works [1], we did not use the VISTA negatives because these so-called negative enhancers were probably real enhancers, and they are not representatives of non-enhancer regions.

### The pipeline of EnhancerDBN

**Fig. 1:**
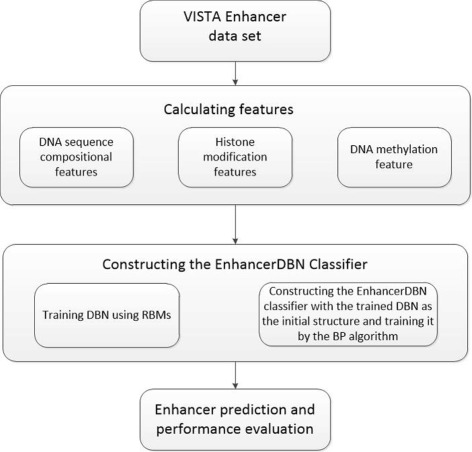
The pipeline of EnhancerDBN

Figure 1 shows the pipeline of the EnhancerDBN method. It consists of three main steps: 1) Feature calculation. Three types of features were used to represent enhancers, including DNA sequence compositional features, histone modifications and DNA methylation. 2) Training the EnhanerDBN classifier for enhancer prediction. A two-step scheme is used. The first step is to construct the DBN by training a series of Restricted Boltzmann Machines (RBMs); the second step is to train and optimize the EnhancerDBN classifier by using the trained DBN and an additional output layer with the backpropagation (BP) algorithm [16]. 3) Enhancer prediction and performance evaluation. 10-fold validation was used to evaluate the proposed method. In what follows, we describe the technical details of the major steps.

### Feature calculation

#### DNA sequence compositional features

We used *k*-mers as the sequence compositional features, with *k* ranging from 2 to 4. For a given *k*, there are at most 4^*k*^ *k*-mers in a DNA sequence. As each DNA fragment can be obtained from either strand of the DNA genome, one *k*-mer and its opposite complement *k*-mer can be regarded as one feature, thus we can reduce the number of sequence compositional features to *N*(*k*)=4^*k*^/2. Take *k* =2 for example, *N*(2)=4^2^/2=8. That is, the number of 2-mer features is 8. Similarly, there are 32 3-mer features, 128 4-mer features. Thus, we have totally 168 *k*-mer features for enhancer representation. For each individual *k*-mer, we counted its frequency in each positive/negative sample sequence and take it as the corresponding feature value.

In addition, we also calculated the total frequency of G and C occurring in each positive/negative sample, and took it as the value of GC content feature.

#### DNA methylation feature

According to previous bioresearch, low DNA methylation was shown to be relevant to enhancers. So we used the level of DNA methylation of each sample as its feature.

The DNA methylation feature was calculated in two steps. First, we obtained the location for each sample in the genome. Then, according to its location, we counted the total value of methylation within the region of the sample, which was used as the sample’s methylation feature.

#### Histone modification features

There are many kinds of histone modifications, including H3K4me1, H3K4me2, H3ac and so forth. Here, we used 106 kinds of histone modifications. Similarly, The histone modification features were calculated in two steps. First, we obtained the location for each positive/negative sample in the genome. Then, according to the location, for each kind of histone modifications, we counted its total amount within the region of the positive/negative sample. Thus, we obtained a 106-dimension histone modification feature vector for each positive/negative sample.

### Constructing the EnhancerDBN classifier

**Fig. 2:**
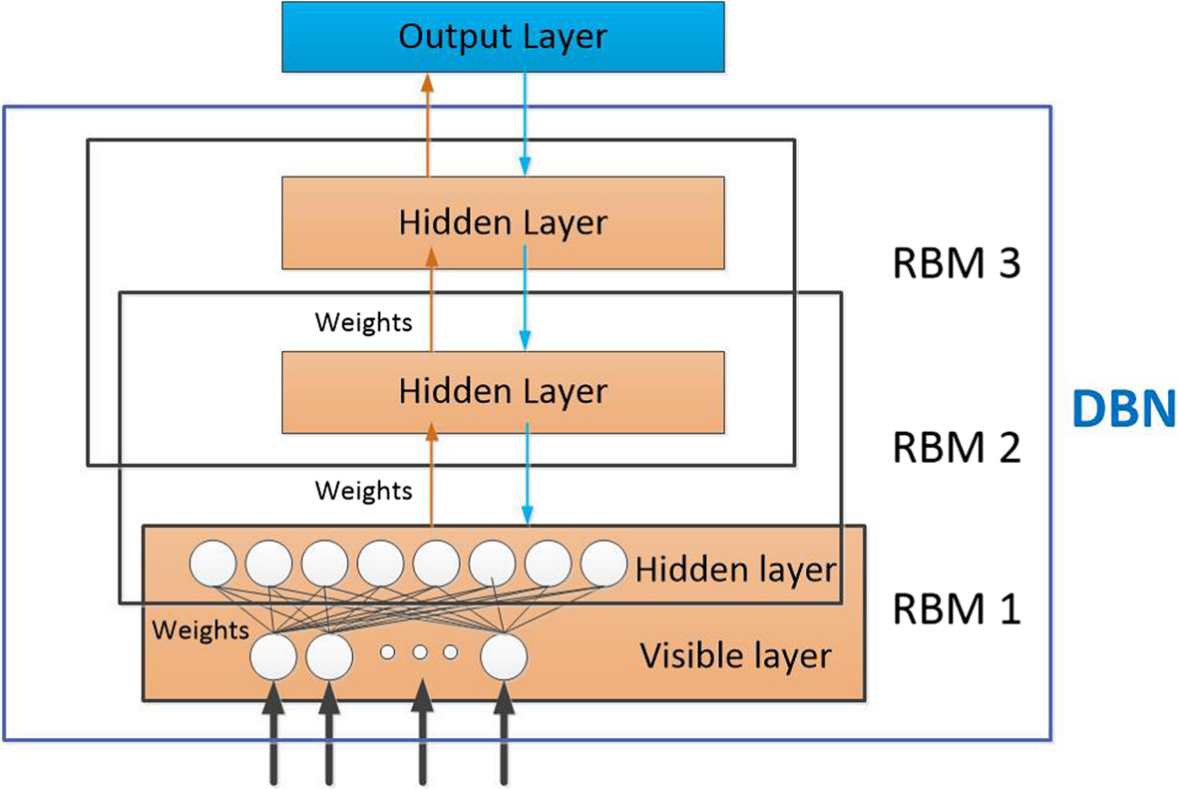
The architecture of the EnhancerDBN classifier

Figure 2 illustrates the architecture of the EnhancerDBN classifier, which consists of a DBN and an output layer. To train the EnhancerDBN classifier, the DBN must be first trained in an unsupervised way. After that, the trained DBN is further combined with the output layer to form a deep neural network (DNN), which is trained by the backpropagation (BP) algorithm in a supervised way, and finally the EnhancerDBN classifier is obtained.

#### Training DBN with RBMs

As shown in Fig. 2, a DBN is a multilayer, stochastic generative model that is constructed by training a stack of RBMs, each of which is trained by using the hidden variables of the previous RBM as its visible variables [16].

Here we built the DBN with 3 RBMs. Each RBM has its own visible layer and output layer. After performance tuning, we set the number of nodes in the hidden layer for the three RBMs to 50, 50 and 200, respectively. As the training samples are 276-dimension vectors, the number of nodes in the visible layer for the 1st RBM is 276. For the 2nd and the 3rd RBMs, the number of nodes in the visible lay is 50. These three connected RBMs construct the DBN with a structure of 276-50-50-200.

A greedy layer-wise unsupervised training process was performed to the DBN with RBMs as its building blocks. The training process is as follows:

- Step 1. Training the 1st RBM by inputting the training data to its visible layer.
- Step 2. Training the 2nd RBM by treating the hidden layer of the 1st RBM as its visible layer.
- Step 3. Training the 3rd RBM by treating the hidden layer of the 2nd RBM as its visible layer.
- Step 4. Building the DBN with weights and biases learned in the three RBMs.

We can see that the RBMs are trained one by one, obtaining the weights between the visible layer and the hidden layer of each RBM, by using contrastive divergence [18, 19]. The details are presented below.

#### Training restricted Boltzmann machine (RBM)

**Fig. 3:**
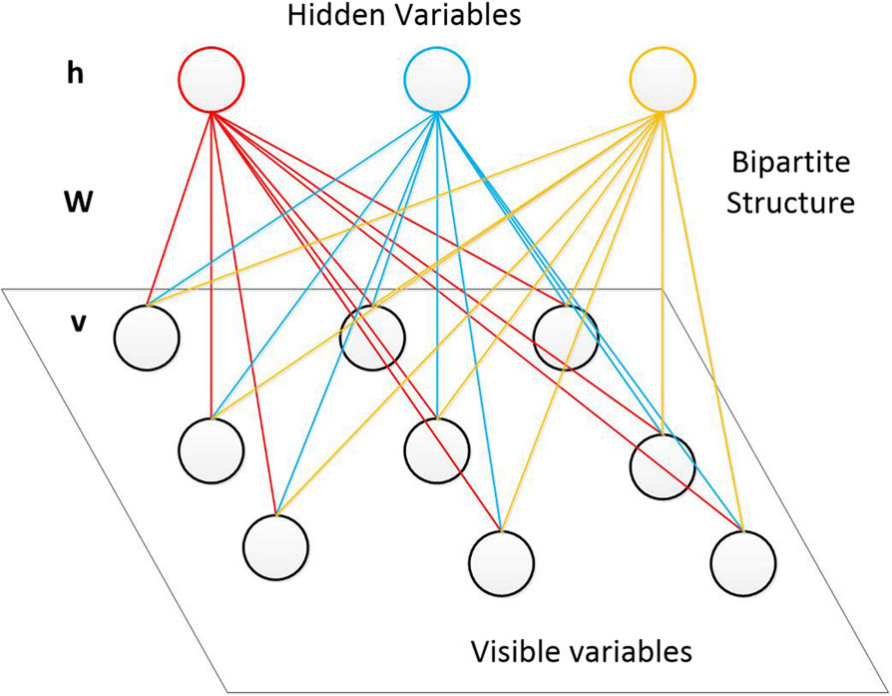
The RBM Architecture

A restricted Boltzmann machine (RBM) is a particular type of random neural network model that has a two-layer architecture as shown in Fig. 3. One layer is called visible layer, which is also the input layer; The other layer is called hidden layer. Nodes in the two layers are fully connected, while there is no connection within the same layer. This constitutes a bipartite structure.

As shown in Fig. 3, the bottom layer contains visible variables (nodes) *v* and the top layer contains hidden variables (nodes) *h*. The matrix *W* is used to represent the symmetric interaction terms between the visible variables and the hidden variables.

The energy function of the joint configuration can be expressed as:

1 $$ E(v,h;\theta)=-\sum_{ij}W_{ij}v_{ij}h_{j}-\sum b_{i}v_{i}-\sum a_{j}h_{j},  $$

where *θ*= {*W,a,b*} represents the model parameters, *a*_*i*_ is the bias of visible unit *i*, and *b*_*j*_ is the bias of hidden unit *j*.

The joint probability distribution of a certain configuration is determined by the Boltzmann distribution (and the energy of this configuration):

2 $$ P_{\theta}(v,h)=\frac{1}{Z(\theta)}exp(-E(v,h;\theta)),  $$

3 $$ Z(\theta)=\sum_{h,v}exp(-E(v,h;\theta)),  $$

where *Z*(*θ*) is the normalization constant.

When a vector *v*= (*v*_1_,*v*_2_,…,*v*_*i*_,…) is input to the visible layer, the binary state *h*_*j*_ of the hidden unit *j* is set to 1 with the probability as follows:

4 $$ P(h_{j}=1|v)=sigmoid\left(\sum_{i}W_{ij}v_{i}+a_{j}\right).  $$

With the states of the hidden units, the binary state *v*_*i*_ of visible unit *i* is set to 1 with the probability below:

5 $$ P(v_{i}=1|h)=sigmoid\left(\sum_{j}W_{ij}h_{j}+b_{i}\right).  $$

A RBM is usually trained as follows:

- Step 1. The states of the visible units are set according to the training data.
- Step 2. Calculating the binary states of the hidden variables by Eq. (4).
- Step 3. After determining the states of all the hidden units, the states of all visible units are determined by Eq. (5).
- Step 4. The gradients of *W* are evaluated by the contrastive divergence (CD) learning algorithm, then the gradient descent algorithm is carrying out to update the parameters *W,a,b*.

#### Training the EnhancerDBN classifier

The DBN is trained in an unsupervised way, which is used to learn features for prediction, and mainly used as the initial network for constructing classifiers.

With the trained DBN above and an additional output layer, our EnhancerDBN classifier was built, and then trained by the same training dataset in a supervised way. The BP algorithm was used to train the classifier. As we employ 10-fold cross validation. We split the data set into ten partitions, with 9 partitions (1334 samples) for training and the rest partitions (containing 148 samples) for test. So 10 trials were done, and the average result was used as the final prediction performance.

## Results and discussion

We conducted 10-fold cross-validation to assess the proposed method. We first evaluated the predictive power of different types of features in terms of prediction error rate, then compared our method with thirteen existing methods in terms of AUC value or prediction accuracy.

### Performance evaluation with different types of features

To evaluate the predictive power of different types of features, we constructed four kinds of feature combinations: “Histone + Sequence”, “Histone + Sequence + GC”, “Histone + Sequence + Methylation” and “Histone + Sequence + Methylation + GC”. Here, “+” means “and”. For example, “Histone + Sequence” means using both sequence compositional features and histone modification features We compared the error rates of our method when using the four different feature combinations, the results are listed in Table 2.

**Table 2:** Prediction error rates when using different feature combinations

Features	Error rate
Histone + Sequence	0.115
Histone + Sequence + GC	0.102
Histone + Sequence + Methylation	0.099
Histone + Sequence + Methylation + GC	0.0915

From Table 2, we can see that when either GC content or DNA methylation is included as feature, the error rate decreases, and when both GC content and DNA methylation are considered, the lowest error rate is achieved. This result shows that GC content and DNA methylation are relevant to enhancers, can serve as effective features for predicting enhancers.

### Performance comparison with existing methods

**Fig. 4:**
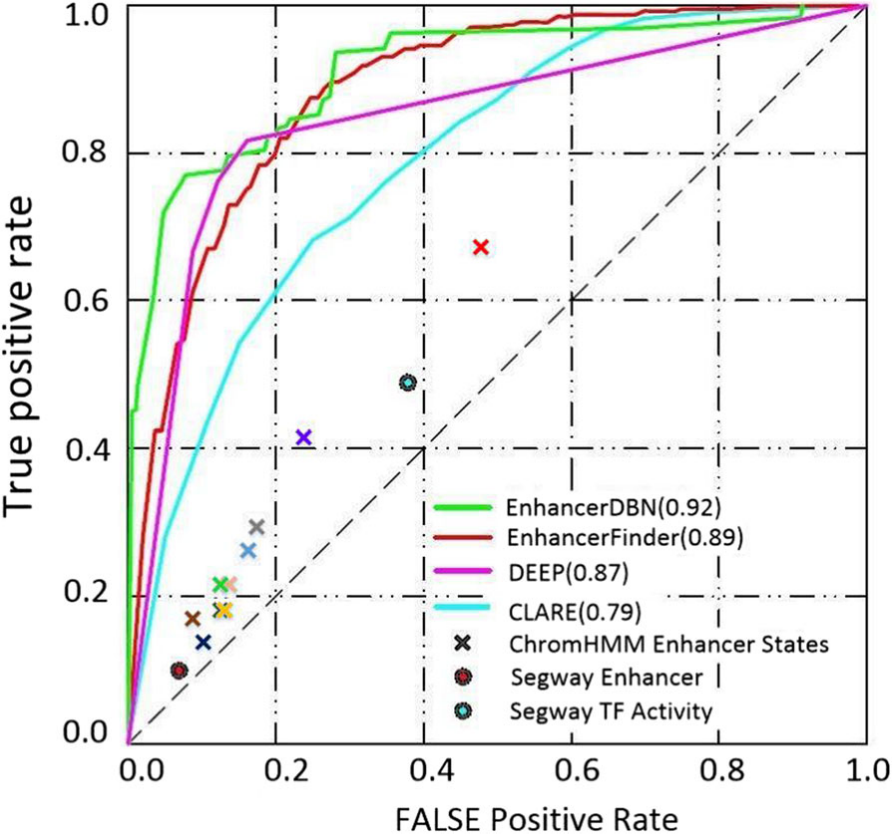
Performance comparison with five typical existing methods in ROC space. The “ ×” of different colors are used for ChromHMM to represent state predictions based on data from different ENCODE cell types: GM12878 (blue), H1-hESC (violet), HepG2 (brown), HMEC (tan), HSMM (gray), HUVEC (light green), K562 (green), NHEK (orange), NHLF (light blue), and all cell types (red)

The EnhancerDBN model was implemented in Matlab by using the DBN algorithm, with the nodes of hidden layers being 50-50-200. The input for the model is the matrix with enhancer samples as rows and features as columns. Here, we first compared our method with five existing methods, including EnhancerFinder [1], CLARE [20], DEEP [21], ChromHMM and Segway in ROC space. Note that comparisons with the existing methods are not easy due to the fact that most existing methods were developed in different contexts. CLARE is a popular method of identifying enhancers using DNA sequence, transcription factor binding site motifs and other sequence patterns, it is publicly available as a web server. The DEEP method and EnhancerFinder work with the VISTA Enhancer Browser. To evaluate ChromHMM and Segway, we considered the states overlapping our training and testing regions. Any region with an overlapping enhancer state was considered an enhancer and the others were non-enhancers. As a result, we obtained a single point in ROC space for the state predictions. Since there is no score or confidence value associated with the state assignments, a full ROC curve could not be obtained for these methods. The results are presented in Fig. 4.

Actually, there are some other methods in the literature. So we then compared our method with eight other existing methods in terms of prediction accuracy, since no confidence values associated with these methods. Table 3 presents the accuracy comparison of our method with the eight existing methods. From this table, we can see that our EnhancerDBN obtains a 92% accuracy, while Chromogens and RFECS both achieve 90.0% accuracy, but the others have only about 80.0% or lower accuracy. So our method is still the best.

**Table 3:** Accuracy comparison with other eight existing methods

Method	Description	Epigenetic feature type	Accuracy(%)	Website	Reference
ChAT	Dynamic Programming	Histone modification	41.7	—	[22]
ChromaSig	Likelihood Function Clustering	Histone modification, Histone distribution	62.6	Bioinformatics- renlab.ucsd.edu/rentrac/wiki/ChromaSig	[23]
CSI-ANN	Artificial Neural Network	Histone modification	66.3	www.medicine.Uiowa.edu/Labs/tan/	[24]
Chromogens	Support Vector Machine	Histone modification	90.0	sysimm.ifrec.saka-u.ac.jp/download/Diego/	[25]
Won’s method	Hidden Markov Model	Histone modification	80.0	nash.ucsd.edu/chromatin.tar.gz.	[26]
BNFinder	Bayes Network	Histone modification, Pol II site	78.0	bioputer.mimuw.edu.pl/software/bnf/	[27]
Yip’s method	Random Forest	Histone modification	67.0	metatracks.Encodenets.Gersteinlab.org/	[28]
RFECS	Random Forest	Histone modification	90.0	enhancer.ucsd.edu/renlab/RFECS_enhancer_prediction/	[12]
EnhancerDBN	DEEP Belief Network	Histone modification	92.0	—	—

In summary, either from the perspective of accuracy or in terms of ROC AUC, EnhancerDBN achieves the best performance, in comparison with totally thirteen existing methods. This result shows that EnhancerDBN is an effective and reliable method to predict enhancers.

## Conclusions

In this study, we proposed EnhancerDBN, an new enhancer predicting method based on DBN. The VISTA Enhancer dataset was used to train and test the proposed method. Three kinds of features, including DNA sequence, histone modifications and DNA methylation were used to represent positive/negative enhancers. EnhancerDBN used a two-step scheme to construct and train a deep neural network (DNN) classifier, which turns the prediction problem into a binary classification task to decide whether or not a DNA region is an enhancer. The first step is to construct a DBN using RBMs, and the second step is to train and optimize the DNN classifier using the BP algorithm. Our experimental results demonstrate that EnhancerDBN outperforms thirteen existing methods, and GC content and DNA methylation are informative for enhancer prediction. In the future, we will explore other deep learning techniques to predict enhancers and other cis-regulatory elements.

## Abbreviations

RBM: Restricted Boltzmann Machines
DBN: Deep Belief Network
